# Sacubitril/Valsartan Improves Autonomic Function and Cardiopulmonary Parameters in Patients with Heart Failure with Reduced Ejection Fraction

**DOI:** 10.3390/jcm9061897

**Published:** 2020-06-17

**Authors:** Francesco Giallauria, Giuseppe Vitale, Mario Pacileo, Anna Di Lorenzo, Alessandro Oliviero, Francesco Passaro, Roberta Calce, Alessandro Parlato, Crescenzo Testa, Giuseppe D’Ambrosio, Giuseppe Romano, Francesco Clemenza, Silvia Sarullo, Elio Venturini, Marco Gentile, Cinzia Nugara, Gabriella Iannuzzo, Antonello D’Andrea, Carlo Vigorito, Filippo M. Sarullo

**Affiliations:** 1 Department of Translational Medical Sciences, Federico II University of Naples, 80131 Naples, Italy; dilorenzoanna2@gmail.com (A.D.L.); alessandro.oliviero0695@gmail.com (A.O.); francescopassaro1996@gmail.com (F.P.); roberta-calce@virgilio.it (R.C.); alessandroparlato96@gmail.com (A.P.); kre.testa@gmail.com (C.T.); giuseppe.dambrosio91@gmail.com (G.D.); vigorito@unina.it (C.V.); 2 Cardiovascular Rehabilitation Unit, Buccheri La Ferla Fatebenefratelli Hospital, 90123 Palermo, Italy; giuseppevit@hotmail.com (G.V.); silvia.sarullo@libero.it (S.S.); cinzianugara@gmail.com (C.N.); sarullo.filippo@fbfpa.it (F.M.S.); 3 Unit of Cardiology and Intensive Care, “Umberto I” Hospital, Viale San Francesco, 84014 Nocera Inferiore (SA), Italy; pacmario@yahoo.it (M.P.); antonellodandrea@libero.it (A.D.); 4 Cardiology Unit and Research Office, Department for the Treatment and Study of Cardiothoracic Diseases and Cardiothoracic Transplantation IRCCS-ISMETT, 90127 Palermo, Italy; romanogiuseppe81@gmail.com (G.R.); fclemenza@ismett.edu (F.C.); 5 Cardiac Rehabilitation Unit, Azienda USL Toscana Nord-Ovest, Cecina Civil Hospital, 57023 Cecina (LI), Italy; vent.elio@tin.it; 6 Department of Clinical Medicine and Surgery, Federico II University, 80131 Naples, Italy; margenti@unina.it (M.G.); gabriella.iannuzzo@unina.it (G.I.); 7 IRCCS Centro Neurolesi Bonino Pulejo Messina, University of Palermo, 90123 Palermo, Italy

**Keywords:** sacubitril/valsartan, heart failure, heart rate recovery, peak oxygen consumption, autonomic function, cardiopulmonary exercise stress testing

## Abstract

Background: Heart rate recovery (HRR) is a marker of vagal tone, which is a powerful predictor of mortality in patients with cardiovascular disease. Sacubitril/valsartan (S/V) is a treatment for heart failure with reduced ejection fraction (HFrEF), which impressively impacts cardiovascular outcome. This study aims at evaluating the effects of S/V on HRR and its correlation with cardiopulmonary indexes in HFrEF patients. Methods: Patients with HFrEF admitted to outpatients’ services were screened out for study inclusion. S/V was administered according to guidelines. Up-titration was performed every 4 weeks when tolerated. All patients underwent laboratory measurements, Doppler-echocardiography, and cardiopulmonary exercise stress testing (CPET) at baseline and at 12-month follow-up. Results: Study population consisted of 134 HFrEF patients (87% male, mean age 57.9 ± 9.6 years). At 12-month follow-up, significant improvement in left ventricular ejection fraction (from 28% ± 5.8% to 31.8% ± 7.3%, *p* < 0.0001), peak exercise oxygen consumption (VO_2peak_) (from 15.3 ± 3.7 to 17.8 ± 4.2 mL/kg/min, *p* < 0.0001), the slope of increase in ventilation over carbon dioxide output (VE/VCO_2 slope_ )(from 33.4 ± 6.2 to 30.3 ± 6.5, *p* < 0.0001), and HRR (from 11.4 ± 9.5 to 17.4 ± 15.1 bpm, *p* = 0.004) was observed. Changes in HRR were significantly correlated to changes in VE/VCO_2slope_ (*r* = −0.330; *p* = 0.003). After adjusting for potential confounding factors, multivariate analysis showed that changes in HRR were significantly associated to changes in VE/VCO_2slope_ (Beta (B) = −0.975, standard error (SE) = 0.364, standardized Beta coefficient (Bstd) = −0.304, *p* = 0.009). S/V showed significant reduction in exercise oscillatory ventilation (EOV) detection at CPET (28 EOV detected at baseline CPET vs. 9 EOV detected at 12-month follow-up, *p* < 0.001). HRR at baseline CPET was a significant predictor of EOV at 12-month follow-up (B = −2.065, SE = 0.354, *p* < 0.001). Conclusions: In HFrEF patients, S/V therapy improves autonomic function, functional capacity, and ventilation. Whether these findings might translate into beneficial effects on prognosis and outcome remains to be elucidated.

## 1. Introduction

Heart failure is characterized by autonomic dysfunction [1,2]. Heart rate recovery (HRR) after graded exercise stress testing is one of the commonly used indexes reflecting autonomic activity [1,2]. HRR is defined as the fall in heart rate during the first minute after exercise and is easily calculated as the difference in heart rate between the peak of exercise and one minute after exercise cessation. HRR is a marker of vagal tone, thanks to sympathetic deactivation and parasympathetic reactivation [1,2]. The first minute of post-exercise recovery is predominantly secondary to parasympathetic reactivation [1,3,4,5]. However, even though vagal-related HRR is parasympathetically mediated, previous studies suggest that it might represent independent aspects, but complementary information, with regard to parasympathetic function [6].

In patients with heart failure (HF), the total sympathetic nervous activity is augmented owing to loss of baroreflex sensitivity and to increased sympathetic afferent activity [7,8,9]. The sympathetic nervous system negatively impacts the cardiovascular system in HF; down-regulation of beta1-receptors and direct toxic effects on the myocardium contribute to cardiac remodeling and life-threatening arrhythmias [7,8,9].

Sacubitril/valsartan (S/V) is a treatment for HF with reduced ejection fraction (HFrEF), which impressively impact cardiovascular prognosis by reducing major adverse cardiovascular events such as cardiovascular mortality and sudden death [10]. Moreover, S/V significantly improves cardiopulmonary functional capacity and ventilatory parameters [11,12,13,14].

The present study aims at evaluating the effects of S/V on autonomic function (as evaluated by HRR) and its correlation with cardiopulmonary indexes in HFrEF patients.

## 2. Experimental Section

### 2.1. Study Population

Patients with HFrEF admitted to outpatients’ services were screened for study inclusion. According to Italian reimbursement criteria, (1) symptomatic HF was defined as New York Heart Association (NYHA) class II–IV; (2) left ventricular ejection fraction (LVEF) ≤35%; (3) previous treatment with maximal tolerated dose for at least 6 months of angiotensin-converting enzyme inhibitor (ACE-Inh) or angiotensin receptor blocker (ARB); (4) systolic blood pressure ≥ 100 mmHg; (5) serum potassium (K^+^) ≤5.4 mmol/L, estimated glomerular filtration rate ≥30 mL/min/1.73 m^2^; (6) absence of severe liver disease (Child–Pugh C); and (7) no history of angioedema, S/V was prescribed when indicated. Patients were encouraged to undergo physical activity (according to patient’s age, past habits, comorbidities, preferences, and goals) as indicated by European guidelines (at least 2.5 h/week of moderate intensity aerobic activity in multiple bouts each lasting ≥10 min, 5 days/week) [15]. Exclusion criteria for study entry were as follows: HF hospitalization within 90 days before ambulatory evaluation, myocardial revascularization within 180 days before ambulatory evaluation, concomitant initiation of cardiac resynchronization therapy and/or percutaneous mitral valve treatment during study follow-up or in the previous 6 months, congenital heart disease, and inability to perform cardiopulmonary exercise stress testing (CPET). After inclusion, HFrEF patients were stratified according to angiotensin receptor neprilysin inhibitor (ARNI) dose (ARNI_1_ = 24/26 mg bid, ARNI_2_ = 49/51 mg bid, and ARNI_3_ = 97/103 mg bid) [16]. Up-titration was performed every 4 weeks when tolerated. Changes in the dosage of diuretics were allowed during the study follow-up if deemed necessary. All patients underwent laboratory measurements, Doppler-echocardiography, and CPET at baseline and at 12-month follow-up.

The Institutional Research Review Boards of the Cardiovascular Rehabilitation Unit of Buccheri La Ferla Fatebenefratelli Hospital and of the Department for the Treatment and Study of Cardiothoracic Diseases and Cardiothoracic Transplantation IRCCS-ISMETT (IRRB/23/15; Palermo, Italy) approved this prospective, observational study. All patients provided informed consent.

### 2.2. Laboratory Measurements

N-terminal pro-brain natriuretic peptides (NT-proBNP) serum levels were detected at baseline and 12-month follow-up as previously described [17,18]. At baseline and after 12 months, fasting blood samples were collected between 08:00 and 09:00, avoiding blood sampling within 24 h of physical activity or CPET. NT-pro-BNP was determined with a sandwich immunoassay on an Elecsys 2010 (Roche diagnostics, Milano, Italy) with analytical range extended from 5 to 35.000 pg/mL.

### 2.3. Doppler Echocardiography

Doppler echocardiography was performed a median of 1 day (range 0–3 days) after study enrollment and at 12-month follow-up. Measurements were obtained at least two times for an average. Standard views were recorded according to guidelines [19]. Indexing body size variables included body surface area (BSA, m^2^), body mass index (BMI, kg/m^2^), and height (cm). Indexed left atrial (LA) volume, indexed LV end-diastolic volume (LVEDV) and indexed LV end-systolic volume (LVESV), LV septal and posterior end-diastolic wall thickness, and LVEF were recorded. Non-indexed LA diameter, with 40 mm as the cut-off for normal, was also assessed [20,21]. Pulsed-wave Doppler echocardiography (before and with Valsalva maneuver) was used to explore mitral inflow from the apical four-chamber view [22]; and diastolic filling was categorized as normal (grade 0), impaired relaxation (grade 1), pseudonormal pattern (grade 2), or restrictive (grade 3) [23]. Global and regional myocardial function was investigated using Tissue Doppler imaging. The same physician, blinded to the patient allocation into the study protocol and unaware of the results of cardiopulmonary exercise stress testing (CPET), performed all Doppler-echocardiography studies.

### 2.4. Cardiopulmonary Exercise Stress Testing

All patients underwent incremental CPET on a bicycle ergometer as previously described [12]. Before each test, calibration of oxygen and carbon dioxide analyzers and of the flow mass sensor was performed using available precision gas mixtures and a 3 L syringe, respectively. In order to stabilize gas measurements, patients remained seated on the ergometer for at least 3 min before starting to pedal.

After a warm-up period (60 s at 0 Watts workload), a ramp protocol of 15 W/min with pedaling kept constant at 55–65 revolutions/min was started and continued until exhaustion. Then, 12-lead electrocardiogram (ECG) was monitored continuously during the test, and cuff blood pressure was manually recorded every 2 min. Breath-by-breath analysis for respiratory gas exchange measurements used computerized metabolic cart (V_max_^®^ 2900 metabolic cart, SensorMedics, Yorba Linda, CA, USA). VO_2peak_ was recorded as the mean value of VO_2_ during the last 20 s of the test and expressed in millilitres per kilogram per minute. At the end of CPET, the primary reason for stopping was identified. Predicted VO_2peak_ was determined by use of a sex-, age-, height-, and weight-adjusted and protocol-specific formula outlined by Wassermann et al. [24]. Two experienced reviewers (G.V. and F.M.S.) assessed ventilatory anaerobic threshold (VAT) with the V-slope method [25]. The ventilation (VE) versus carbon dioxide production (VCO_2_) relationship was measured by plotting ventilation (VE, l/min) against carbon dioxide production (VCO_2_, L/min) obtained every 10 s of exercise (VE/VCO_2slope_). The VE/VCO_2slope_ was calculated as a linear regression function, excluding the non-linear part of the relationship after the onset of acidotic drive to ventilation. Exercise oscillatory ventilation (EOV), a cyclic fluctuation of minute ventilation and expired gas kinetics occurring during exercise, was reported when identified [26]. The physician who will perform CPET will be unaware of the results of blood sampling and Doppler echocardiography.

### 2.5. Statistics

Descriptive statistics are given in terms of means ± standard deviation or in percentage for nominal variables. Χ^2^ test was used for contingency tables. Within-group changes in the reported variables were evaluated by the paired t-test or Wilcoxon signed rank test for non-normally distributed variables. Unpaired t-test and Mann–Whitney rank sum test were used for between-group comparisons. Differences between groups and changes over time within each group (time effect) as well as any interaction (different trends over time between groups) were assessed by two-way repeated measures analysis of variance (ANOVA). The bivariate correlations procedure was used to compute Pearson or Spearman correlation coefficients with the significance levels. Multiple linear regression analysis was used to test the association between changes in HRR and cardiopulmonary indexes. Models adjusted for potential confounding factors based upon a review of the relevant literature, including age, NYHA class, diabetes, atrial fibrillation, cardiac resynchronization therapy (CRT)/pacemaker, drug therapy (beta-blockers, ivabradine, and loop diuretics use, separately), NT-proBNP levels, maximal ARNI dose reached, and changes in VO_2peak_ and changes in VE/VCO_2slope_. Logistic regression analysis was used for testing the association between HRR evaluated at baseline CPET and exercise oscillatory ventilation detected at 12-months follow-up CPET. A *p* value < 0.05 was considered statistically significant. All analyses were performed by SPSS version 23.0 (SPSS Inc., Chicago, IL, USA).

## 3. Results

The study population consisted of 134 HFrEF patients (87% male, mean age 57.9 ± 9.6 years). Baseline clinical and demographic characteristics of the study population and stratified by ARNI dose are given in Table 1 and Table 2, respectively. Overall, mean LVEF was 28.1% ± 5.9% and, at baseline, the majority of patients had NYHA class II (62%) or class III (34%), whereas no patients had NYHA class IV (Table 1). At 12-month follow-up, a significant improvement in NYHA class was observed: 104 patients (77.2%) had NYHA class II and 15 patients (11.4%) had NYHA class III (Table 1).

Major comorbidity was represented by hypertension (54%), dyslipidemia (59%), diabetes (34%), chronic obstructive pulmonary disease (COPD) (16%), and atrial fibrillation (16%) (Table 1). At study entry, 6 patients (4.5%) had pacemaker (PMK), 102 patients (76%) had PMK plus implantable cardioverter-defibrillator (ICD), and 23 patients (17%) had cardiac resynchronization therapy (CRT) devices (Table 1). At 12-month follow-up, 103 patients (77%) had PMK-ICD and a total of 25 (18%) patients had CRT devices. Forty-four patients were active smokers (Table 1).

Clinical and demographic characteristics of the study population stratified by ARNI dose are given in Table 3 At study entry, 90 (67.2%) HFrEF patients were taking ARNI_1_ dose, 40 (31.3%) HFrEF patients were taking ARNI_2_ dose, and 2 (1.5%) HFrEF patients were taking ARNI_3_ dose; whereas at 12-month follow-up, 39 (29.1%) HFrEF patients were taking ARNI_1_ dose, 48 (35.8%) HFrEF patients were taking ARNI_2_ dose, and 47 (35.1%) HFrEF patients were taking ARNI_3_ dose.

Baseline and 12-month follow-up Doppler-echocardiography parameters in overall population and stratified by ARNI dose are reported in Table 3 and Table 4, respectively. A significant improvement in LVEF was observed at 12-month follow-up (from 28% ± 5.8% to 31.8% ± 7.3%, *p* < 0.0001) (Table 3). LVEF improvement was greater among patients treated with higher ARNI doses (from 28.4% ± 6.2% to 29.2% ± 7.2%, *p* = 0.415 in ARNI_1_ group; from 27.4% ± 6.4% to 32.1% ± 6.5%, *p* = 0.006 in ARNI_2_ group; and from 28.2% ± 4.9% to 33.8% ± 8.0%, *p* = 0.001 in ARNI_3_ group).

**Figure 1:**
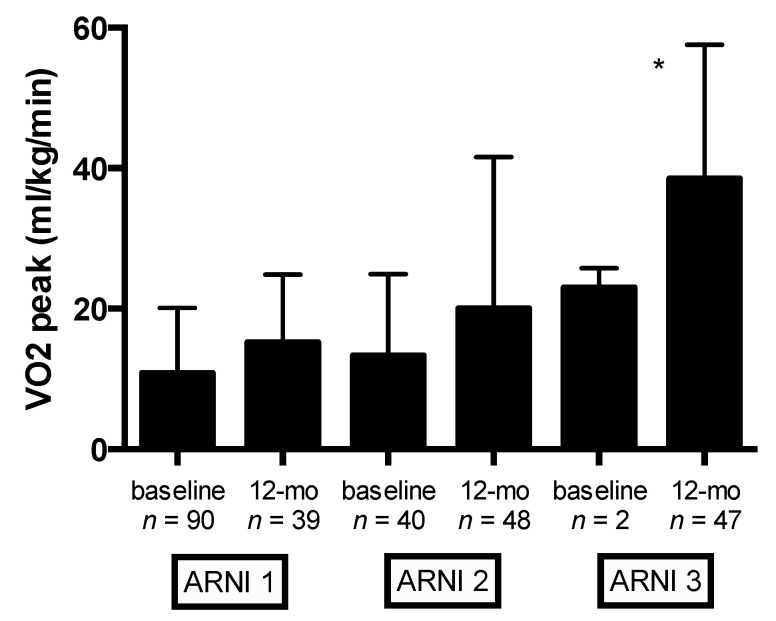
Baseline and 12-month follow-up values of VO2 peak (mL/Kg/min) stratified by ARNI dose. Captions: angiotensin receptor neprilysin inhibitor (ARNI) dose (ARNI_1_ = 24/26 mg bid, ARNI_2_ = 49/51 mg bid, and ARNI_3_ = 97/103 mg bid); peak oxygen consumption (VO2_peak_). * *p <* 0.0001 (ANOVA).

Baseline and 12-month follow-up CPET parameters in the overall population and stratified by ARNI dose are reported in Table 5 and Table 6, respectively. Overall, S/V therapy exerted beneficial effects on cardiopulmonary functional capacity (Table 5). Significant changes in VO_2peak_ and predicted VO_2peak_ (%) were observed at 12-month follow-up (from 15.3 ± 3.7 to 17.8 ± 4.2 mL/kg/min, *p* < 0.0001; and from 56.4% ± 13.9% to 64.8% ± 17.8%, *p* < 0.0001, respectively) (Table 5). Mean VO_2peak_ (mL/kg/min) values according to ARNI dose at baseline and 12-month follow-up are shown in Figure 1. Compared with the lowest ARNI treatment dose, HFrEF patients receiving the higher ARNI dose (97/103 mg bid) had greater VO_2peak_ improvement (*p* < 0.0001) (Figure 1). Compared with baseline, significant improvement in other cardiopulmonary functional capacity parameters were observed at 12-month follow-up: maximal workload and ΔVO2/Δwork significantly improved from 72.8 ± 25.1 to 90.5 ± 28.1 watts, *p* < 0.0001, and from 9.2 ± 1.6 to 10.2 ± 1.5 mL/min/watt, *p* < 0.0001, respectively (Table 5).

**Figure 2:**
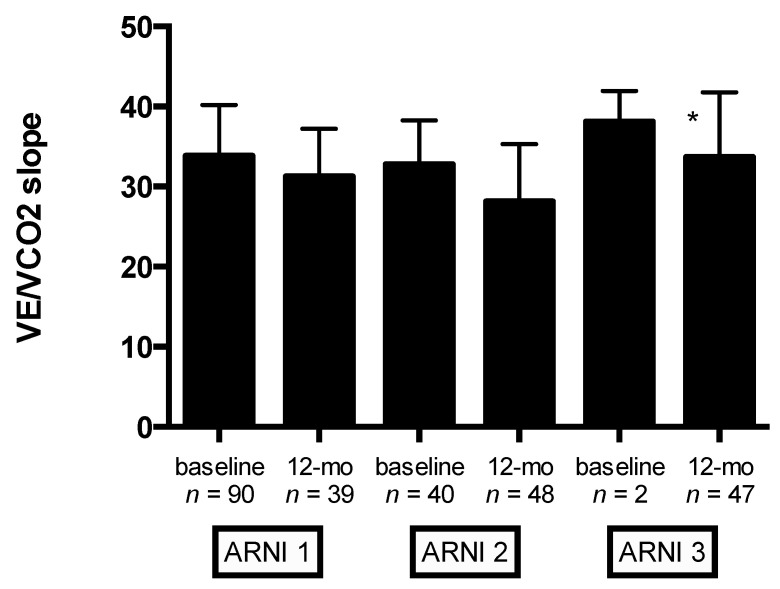
Baseline and 12-month follow-up values of VE/VCO_2slope_ stratified by ARNI dose. Captions: angiotensin receptor neprilysin inhibitor (ARNI) dose (ARNI_1_ = 24/26 mg bid, ARNI_2_ = 49/51 mg bid, and ARNI_3_ = 97/103 mg bid); the slope of increase in ventilation over carbon dioxide output (VE/VCO2slope); * *p* < 0.0001 (ANOVA).

Moreover, significant improvement in VE/VCO_2slope_ was observed at 12-month follow-up (from 33.4 ± 6.2 to 30.3 ± 6.5, *p* < 0.0001) (Table 5). Mean changes in VE/VCO_2slope_ according to ARNI dose at baseline and 12-month follow-up are shown in Figure 2. Compared with the lowest ARNI treatment dose, HFrEF patients receiving the higher ARNI dose (97/103 mg bid) had greater VE/VCO_2slope_ improvement (*p* < 0.0001) (Figure 2). Notably, significant improvement in other ventilatory parameters was observed at 12-month follow-up: peak ventilation and respiratory rate significantly increase from baseline (from 48.1 ± 12.3 to 61.4 ± 18.9 L/min, *p* < 0.0001; and from 30.8 ± 6.3 to 33.6 ± 7.4, *p* < 0.0001, respectively) (Table 5). In addition, HFrEF patients treated with S/V showed a significant reduction in EOV detection at CPET (28 EOV detected at baseline CPET vs. 9 EOV detected at 12-month follow-up, *p* < 0.001). HRR at baseline CPET was a significant predictor of EOV at 12-month follow-up (B = −2.065, SE = 0.354, *p* < 0.001).

**Figure 3:**
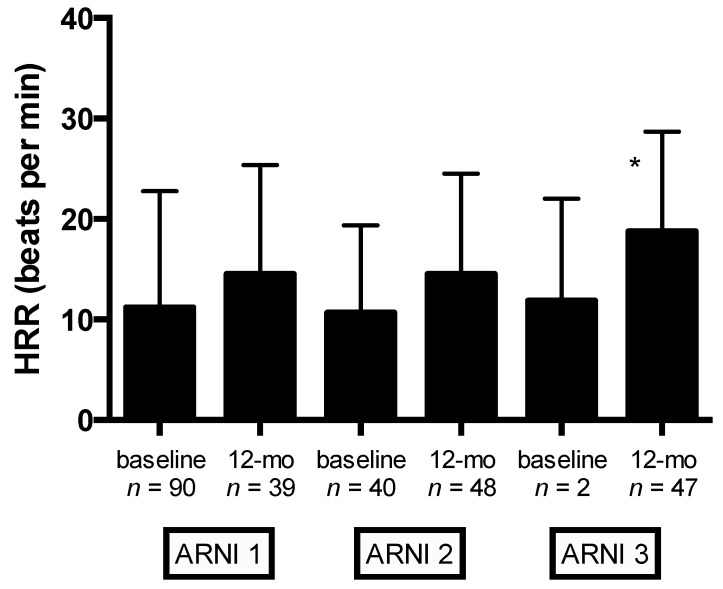
Baseline and 12-month follow-up values of HRR stratified by ARNI dose. Captions: angiotensin receptor neprilysin inhibitor (ARNI) dose (ARNI_1_ = 24/26 mg bid, ARNI_2_ = 49/51 mg bid, and ARNI_3_ = 97/103 mg bid); HRR, heart rate recovery; * *p* = 0.009.

Significant changes in autonomic function (as evaluated by HRR) were observed at 12-month follow-up (from 11.4 ± 9.5 to 17.4 ± 15.1 bpm, *p* = 0.004) (Table 5). Mean changes in HRR stratified by ARNI dose at baseline and 12-month follow-up are shown in Figure 3. Compared with the lowest ARNI treatment dose, HFrEF patients receiving the higher ARNI dose (97/103 mg bid) had greater HRR improvement (*p* = 0.009) (Figure 3).

**Figure 4:**
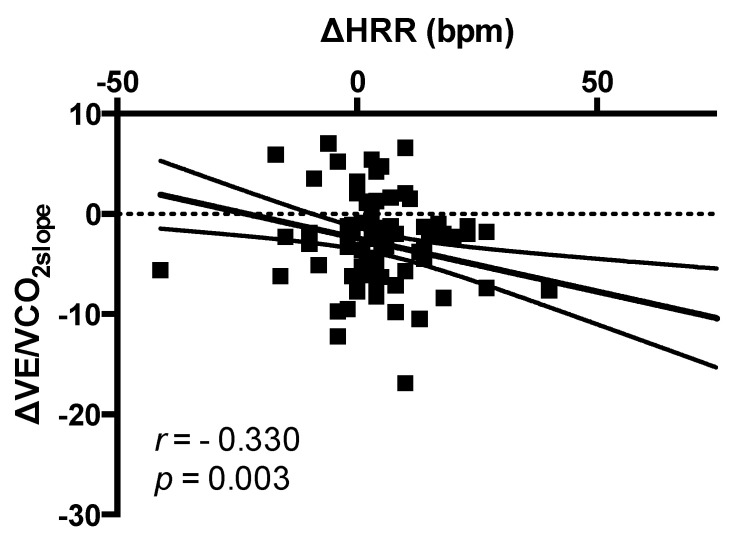
Correlation between changes in HRR and changes in VE/VCO_2slope._ Captions: HRR: heart rate recovery; VE/VCO_2slope_: the slope of increase in ventilation over carbon dioxide output.

No correlation between changes in HRR (bpm) and changes in systolic blood pressure (SBP) (mmHg) was found (*r* = −0.022, *p* = 0.850). Changes in HRR (bpm) were significantly correlated to changes in VE/VCO_2slope_ (*r* = −0.330; *p* = 0.003) (Figure 4). After adjusting for age, NYHA class, diabetes, atrial fibrillation, CRT/pacemaker, NT-proBNP levels, drug therapy (beta-blockers, ivabradine, and loop diuretics use), maximal ARNI dose reached, and changes in VO_2peak_, multivariate analysis showed that changes in HRR were significantly associated to changes in VE/VCO_2slope_ (B = −0.975, SE = 0.364, Bstd = −0.304, *p* = 0.009).

Finally, at a median follow-up of 26 ± 6.7 months, the mortality rate was 10% (13 cardiovascular deaths), and 42 HF hospitalizations occurred. No significant association between ARNI dose or changes in HRR and these hard endpoints was found.

## 4. Discussion

This study showed a significant improvement in autonomic function as measured by HRR in HFrEF patients undergoing S/V treatment for 1 year. Autonomic function ameliorating, as expressed by the increase in HRR, was associated to the improvement in cardiopulmonary function.

To the best of our knowledge, this is the first study reporting beneficial effects on autonomic function in HFrEF patients undergoing S/V therapy. Previous studies showed that autonomic function is favorably improved by structured exercise training in patients after acute myocardial infarction or chronic heart failure [27,28,29,30]. However, exercise-induced improvement in cardiopulmonary and autonomic function is lost when exercise sessions is abandoned [28]. In a recent meta-analysis including patients with coronary artery disease, exercise-based cardiac resynchronization (CR) improved post-exercise parasympathetic function, with greater effects in younger patients with coronary artery disease and in patients who have undergone percutaneous intervention [30]. Notably, the overall effect size showed significant differences in HRR (MD = +5.35; 95% confidence interval (CI) = 4.08–6.61 bpm) in favor of the exercise-based CR group [30].

In this study, HFrEF patients on S/V therapy showed a significant increase in HRR (+6.0 beats/min, *p* = 0.004) that could likely be ascribed to the effect of S/V therapy. Future studies are encouraged in order to investigate whether combined strategies (S/V therapy plus structured exercise training) might improve outcome in HFrEF patients.

HRR is a powerful predictor of all-cause mortality in patients with chronic heart failure [31,32,33,34]. The mechanisms involved in mortality reduction are probably multifactorial, and likely include a central mechanism activated by the release of inhibitory commands from the motor cortex to the parasympathetic center [35], or afferent stimulation from baroreflex or chemoreflex functions [36]. Imai et al. [37] demonstrated that a release of inhibitory central command rather than baro- or chemo-receptor stimulation may play a key role in vagal reactivation after exercise, because the heart rate response early during recovery (i.e., the first 30 s) minimally depends on exercise intensity.

Both enhanced post-infarction inflammatory response [38,39] and autonomic dysfunction [40,41,42,43] are associated with LV remodelling and poor clinical outcomes. Postinfarction healing is a highly regulated process. When post-infarction inflammatory response is efficient, a scar with tensile strength is properly formed, preventing the expansion of the infarction area, with consequent lower incidence of decompensating heart failure. Despite the key role of the healing process in post-infarction LV remodeling, the mechanisms underlying the initiation and regulation of these processes remain to be elucidated. Experimental models showed that S/V attenuates cardiac remodeling and dysfunction after myocardial infarction by reducing cardiac fibrosis and hypertrophy [44,45]. The same mechanicistic pathways might be at the basis of the favorable effect of S/V therapy on cardiac remodeling and inflammatory status in HFrEF patients and, consequently, responsible for the impressive outcome improvement [10].

Although this cohort of HFrEF patients did not undergo structured exercise-based cardiac rehabilitation programs, they were encouraged to maintain an adequate level of physical activity according to guidelines. It is possible that S/V therapy by ameliorating exercise tolerance could favor the engagement of leisure time physical activity with a potential protective beneficial effect on arrhythmias and mortality. The PARADIGM-HF trial [10] showed a reduced risk for sudden death (HR = 0.80, 95% CI = 0.68–0.94, *p* = 0.008) in HFrEF patients treated with S/V. Because only 15% of HF patients from PARADIGM-HF trial in both arms had implantable cardioverter-defibrillator (ICD), it could be hypothesized that the improvement in sudden death could be related to the improvement in autonomic function and its related protective antiarrhytmic effect. In our cohort, 76% of HFrEF patients had ICD and no major arrhythmic episodes were detected at device check.

EOV, a cyclic fluctuation of minute ventilation and expired gas kinetics occurring during exercise, is a marker of HF severity, and predicts either mortality or morbidity [26]. Interestingly, S/V showed a significant reduction in EOV detection at CPET (28 EOV detected at baseline CPET vs. 9 EOV detected at 12-month follow-up, *p* < 0.001). It could be hypothesized that the beneficial effect on autonomic function may translate to a more efficient and coordinated ventilation pattern. Future studies are eagerly awaited in order to establish whether EOV improvement might improve prognosis in HFrEF patients treated with S/V.

The relatively small sample size of patients, predominantly adult males, might limit the conclusion of the study. In addition, HRR is only an indirect, although widely accepted, reflection of sympathovagal balance. Furthermore, this study had no statistical power to analyze the relationship between HRR and prognosis. Conversely, the present study for the first time reported beneficial effects on autonomic function (as measured by HRR) in HFrEF patients undergoing S/V therapy.

## 5. Conclusions

In conclusion, 1-year S/V therapy improves autonomic function, functional capacity, and ventilatory pattern in HFrEF patients. Whether these findings might translate into beneficial effects on prognosis and outcome remains to be elucidated.

## Figures and Tables

**Table 1:** Clinical and demographic characteristics of the study population at baseline and 12-month follow-up.

	Baseline	12-Month Follow-Up	
Age (Years)	57.9 ± 9.6		
Gender (Male) (%)	87		
BMI (kg/m^2^)	28.3 ± 4.5	28.2 ± 4.6	0.935
NT-proBNP (pg/mL)	1443.2 ± 1323	1041.2 ± 1171.3	0.015
eGFR (MDRD) (mL/min/1.73 m^2^)	73.1 ± 36.3	76.8 ± 38.8	0.518
Haemoglobin (g/dL)	12.8 ± 1.4	14.2 ± 2.5	0.388
Na^+^ (mEq/L)	139.2 ± 2.6	140 ± 2.3	0.06
K^+^ (mEq/L)	4.2 ± 0.5	4.3 ± 0.5	0.691
SBP (mmHg)	117.1 ± 16.9	102.9 ± 13.4	<0.0001
DBP (mmHg)	71.8 ± 9.7	64.2 ± 6.9	<0.0001
HR (bpm)	68.2 ± 11.5	67.1 ± 8.9	0.435
NYHA Class I	5 (4%)	15 (11.4%) **	<0.0001
NYHA Class II	82 (62%)	104 (77.2%)	
NYHA Class III	46 (34%)	15 (11.4%)	
NYHA Class IV			
Pacemaker (*n*,%)	6 (4.5%)		
CRT (*n*,%)	23 (17%)		
PMK-ICD (*n*,%)	102 (76%)		
Hypertension (*n*,%)	72 (54%)		
Diabetes (*n*,%)	45 (34%)		
Dyslipidemia (*n*,%)	78 (59%)		
Atrial Fibrillation (*n*,%)	22 (16%)		
COPD (*n*,%)	21 (16%)		
Active Smokers	58 (44%)		
*Drug therapy*			
Beta-Blockers (*n*,%)	125 (93%)	125 (93%)	1.00
ACE-Inhibitors * (*n*,%)	81 (60%)		
ARBs * (*n*,%)	29 (21%)		
Loop Diuretics (*n*,%)	111 (83%)	103 (77%)	0.04
Loop Diuretics Dose (mg/day)	87 ± 105	80 ± 97	0.586
MRA (*n*,%)	110 (82%)	109 (81%)	0.533
MRA dose (mg/day)	26 ± 12	24 ± 9	0.174
Ivabradine (*n*,%)	25 (19%)	25 (19%)	1.00
Statin (*n*,%)	97 (72%)	95 (70%)	1.00
ARNI_1_ (*n*,%)	90 (67.2%)	39 (29.1%) **	<0.0001
ARNI_2_ (*n*,%)	40 (31.3%)	48 (35.8%)	
ARNI_3_ (*n*,%)	2 (1.5%)	47 (35.1%)	

Captions: ACE-inhibitors: angiotensin converting enzyme inhibitors (* prior to ARNI therapy); ARBs: angiotensin receptor blockers (* prior to ARNI therapy); ARNI_1_: angiotensin receptor neprilysin inhibitor (ARNI) dose of 24/26 mg bid at baseline; ARNI2: ARNI dose of 49/51 mg bid at baseline; ARNI_3_: ARNI dose of 97/103 mg bid at baseline; BMI: body mass index; COPD: chronic obstructive pulmonary disease; CRT: cardiac resynchronization therapy; DBP: diastolic blood pressure (at the time of cardiopulmonary exercise stress testing (CPET)); eGFR (MDRD): estimated glomerular filtration rate; HR: heart rate; ICD: implantable cardioverter-defibrillator; MRA: mineral corticoid receptor antagonists; NT-proBNP: N-terminal pro-brain natriuretic peptide; NYHA: New York Heart Association; PMK: pacemaker; SBP: systolic blood pressure (at the time of CPET). ** Χ^2^ test was used for contingency tables.

**Table 2:** Clinical and demographic characteristics of the study population stratified by ARNI dose.

	ARNI_1_		ARNI_2_		ARNI_3_	
	Baseline (*n* = 90)	12-Month Follow-Up (*n* = 39)	Baseline (*n* = 40)	12-Month Follow-Up (*n* = 48)	Baseline (*n* = 2)	12-Month Follow-Up (*n* = 47)
Age (Years)	58 ± 9	59 ± 9	57.8 ± 10.8	58.3 ± 11.0	55.5 ± 13.4	56.7 ± 8.4
BMI (kg/m^2^)	27.2 ± 3.9	26.9 ± 4.0	30.4 ± 5.1	28.8 ± 4.7	31.4 ± 2.3	28.6 ± 4.9
NT-proBNP (pg/mL)	1450.15 ± 1466.52	1343.55 ± 1373.42	1390.70 ± 991.90	1151.68 ± 1303.11	2146 ± 11.49	673.3 ± 704.7 *
eGFR (MDRD) (mL/min/1.73 m^2^)	73.3 ± 39.2	69.3 ± 23.7	72.5 ± 31.1	82.2 ± 55.0	77.5 ± 15.9	75.6 ± 13.9
Haemoglobin (g/dL)	13.0 ± 1.6	14.2 ± 2.5	12.0 ± 1.1	12.8 ± 1.4	12.3 ± 1.8	12.7 ± 1.6
Na^+^ (mEq/L)	139.2 ± 2.6	139.8 ± 3.2	139.5 ± 2.6	140.1 ± 1.8	136 ± 0.3	140.4 ± 2.2
K^+^ (mEq/L)	4.2 ± 0.43	4.4 ± 0.60	4.3 ± 0.54	4.2 ± 0.36	4.5 ± 0.32	4.3 ± 0.53
SBP (mmHg)	114.9 ± 14.5	96.9 ± 11.5 *	121.8 ± 20.2	102.9 ± 12.0 *	122.5 ± 31.8	107.9 ± 14.5
DBP (mmHg)	71.3 ± 9.6	62.4 ± 6.7 *	72.7 ± 10.2	63.9 ± 6.6 *	77.5 ± 3.5	65.9 ± 7.2 *
HR (bpm)	67.8 ± 10.5	67.3 ± 7.016	68.0 ± 12.5	67.4 ± 8.6	86.5 ± 26.2	66.6 ± 10.9 *

Captions: * *p* < 0.001 vs. baseline; ARNI_1_: angiotensin receptor neprilysin inhibitor (ARNI) dose of 24/26 mg bid at baseline; ARNI2: ARNI dose of 49/51 mg bid at baseline; ARNI_3_: ARNI dose of 97/103 mg bid at baseline; BMI: body mass index; DBP: diastolic blood pressure (at the time of cardiopulmonary exercise stress testing (CPET)); eGFR (MDRD): estimated glomerular filtration rate; HR: heart rate; NT-proBNP: N-terminal pro-brain natriuretic peptide; SBP: systolic blood pressure (at the time of CPET).

**Table 3:** Baseline and 12-month follow-up Doppler-echocardiography parameters in overall population.

Doppler-Echocardiography Parameters	Baseline	12-Month Follow-Up	*p-*Value
LVEDD (mm)	63.1 ± 6.7	63.6 ± 6.5	0.701
LVEDV (mL)	216.2 ± 56.3	208.7 ± 59.1	0.482
LVEDVi (mL/m^2^)	112.2 ± 27.9	112.9 ± 25.8	0.897
LVEF (%)	28 ± 5.8	31.8 ± 7.3	<0.0001
LAV (mL)	96.7 ± 35.8	86.1 ± 29.3	0.098
LAVi (mL/m^2^)	49.6 ± 17.9	45.6 ± 14.7	0.226
E-Wave (cm/s)	0.92 ± 0.43	0.85 ± 0.42	0.434
A-Wave (cm/s)	0.67 ± 0.31	0.79 ± 0.31	0.094
E/A Ratio	1.53 ± 1.4	1.01 ± 0.74	0.02
Deceleration Time (ms)	175.9 ± 53.8	197.2 ± 59.4	0.113
E’ (cm/s)	0.05 ± 0.02	0.06 ± 0.02	0.047
E/E’ ratio	15.1 ± 6.8	13.4 ± 7.0	0.298
TAPSE (mm)	19.5 ± 4.2	19.3 ± 3.9	0.398

Captions: A-wave: peak velocity flow in late diastole caused by atrial contraction; E-wave: peak velocity blood flow from left ventricular relaxation in early diastole; E’-wave: early diastolic myocardial relaxation; LAV: left atrium volume; LAVi: left atrium indexed volume; LVEDD: left ventricular end-diastolic diameter; LVEDV: left ventricular end-diastolic volume; LVEDVi: left ventricular end-diastolic indexed volume; LVEF: left ventricular ejection fraction; TAPSE: tricuspid annular plane excursion.

**Table 4:** Baseline and 12-month follow-up Doppler-echocardiography parameters in overall population stratified by ARNI dose.

	ARNI_1_		ARNI_2_		ARNI_3_	
	Baseline (*n* = 90)	12-Month Follow-Up (*n* = 39)	Baseline (*n* = 40)	12-Month Follow-Up (*n* = 48)	Baseline (*n* = 2)	12-Month Follow-Up (*n* = 47)
LVEDD (mm)	62.8 ± 6.4	64.3 ± 6.1	63.8 ± 7.7	65.1 ± 5.4	68.0 ± 2.8	61.4 ± 7.6
LVEDV (mL)	210.9 ± 57.7	210.7 ± 53.1	224.3 ± 51.5	213.6 ± 67.2	281.0 ± 41	203.3 ± 64.1
LVEDVi (mL/m^2^)	112.7 ± 28.4	110.9 ± 25.7	107.9 ± 26.6	115.9 ± 21.4	140.5 ± 13.4	113.9 ± 29.9
LVEF (%)	28.4 ± 6.2	29.2 ± 7.2	27.4 ± 6.4	32.1 ± 6.5 *	28.2 ± 4.9	33.8 ± 8.0
LAV (mL)	92.7 ± 35.0	90.8 ± 27.6	106.4 ± 38.4	86.7 ± 37.1	107.0 ± 18.4	79.8 ± 27.4
LAVi (mL/m^2^)	49.1 ± 19.2	48.9 ± 16.3	50.9 ± 14.7	44.3 ± 14.4	53 ± 4.2	41.6 ± 12.6
E-Wave (cm/s)	0.90 ± 0.43	0.97 ± 0.46	1.01 ± 0.44	0.8 ± 0.23	0.65 ± 0.03	0.72 ± 0.45
A-Wave (cm/s)	0.64 ± 0.30	0.71 ± 0.28	0.75 ± 0.33	0.85 ± 0.42	0.85 ± 0.04	0.83 ± 0.22
E/A Ratio	1.6 ± 1.5	1.36 ± 1.44	0.89 ± 0.55	0.94 ± 0.31	0.76 ± 0.035	0.87 ± 0.34
Deceleration Time (ms)	172.5 ± 55.1	191.8 ± 55.9	193.2 ± 47.1	185.0 ± 55.9	143.5 ± 61.5	212.4 ± 67.9
E’ (cm/s)	0.05 ± 0.02	0.06 ± 0.01	0.06 ± 0.01	0.06 ± 0.02	0.05 ± 0.01	0.06 ± 0.02
E/E’ ratio	15.2 ± 7.5	13.2 ± 4.6	15.3 ± 4.6	13.5 ± 5.7	13.3 ± 3.3	13.6 ± 10.4
TAPSE (mm)	19.0 ± 4.1	17.6 ± 3.1	20.8 ± 4.7	19.6 ± 3.3	18.5 ± 0.7	20.0 ± 3.3

Captions: * *p* = 0.01 vs. baseline; A-wave: peak velocity flow in late diastole caused by atrial contraction; ARNI_1_: angiotensin receptor neprilysin inhibitor (ARNI) dose of 24/26 mg bid; ARNI2: ARNI dose of 49/51 mg bid; ARNI_3_: ARNI dose of 97/103 mg bid; E-wave: peak velocity blood flow from left ventricular relaxation in early diastole; E’-wave: early diastolic myocardial relaxation; LAV: left atrium volume; LAVi: left atrium indexed volume; LVEDD: left ventricular end-diastolic diameter; LVEDV: left ventricular end-diastolic volume; LVEDVi: left ventricular end-diastolic indexed volume; LVEF: left ventricular ejection fraction; TAPSE: tricuspid annular plane excursion.

**Table 5:** Baseline and 12-month follow-up cardiopulmonary parameters in overall population.

CPET Parameters	Baseline	12-Month Follow-Up	*p* Value
VO_2peak_ (mL/kg/min)	15.3 ± 3.7	17.8 ± 4.2	<0.0001
Predicted VO_2peak_ (%)	56.4 ± 13.9	64.8 ± 17.8	<0.0001
Watt Max (W)	72.8 ± 25.1	90.5 ± 28.1	<0.0001
VE/VCO_2slope_	33.4 ± 6.2	30.3 ± 6.5	<0.0001
RER	1.14 ± 0.12	1.14 ± 0.15	0.836
HR Rest (bpm)	72.4 ± 10.8	68.9 ± 12.1	0.329
HR Peak (bpm)	106.6 ± 18.2	110.9 ± 20.6	0.001
HRR (bpm)	11.4 ± 9.5	17.4 ± 15.1	0.004
AT-VO_2_ (mL/kg/min)	11.4 ± 2.9	12.1 ± 3.6	0.015
AT-VO_2_ (%)	40.9 ± 11.5	55.5 ± 84.6	0.173
AT-Watt (W)	56.1 ± 24.8	58.5 ± 22.1	0.536
O_2_-Pulse (mL/beat)	11.4 ± 3	13.7 ± 4.6	<0.0001
ΔVO_2_/Δwork (mL/min/watt)	9.2 ± 1.6	10.2 ± 1.5	<0.0001
Peak Ventilation (L/min)	48.1 ± 12.3	61.4 ± 18.9	<0.0001
TIDAL Volume (mL/kg)	1.5 ± 0.38	2.2 ± 3.7	0.114
Respiratory Rate (breaths/min)	30.8 ± 6.3	33.6 ± 7.4	<0.0001

Captions: AT: anaerobic threshold; RER: respiratory exchange ratio; VE: ventilation; VE/VCO_2slope_: the slope of increase in ventilation over carbon dioxide output; VO_2_: oxygen consumption; HR: heart rate; HRR: heart rate recovery; CPET: cardiopulmonary exercise stress testing.

**Table 6:** Baseline and 12-month follow-up cardiopulmonary parameters in study population stratified by ARNI dose.

	ARNI_1_		ARNI_2_		ARNI_3_	
	Baseline (*n* = 90)	12-Month Follow-Up (*n* = 39)	Baseline (*n* = 40)	12-Month Follow-Up (*n* = 48)	Baseline (*n* = 2)	12-Month Follow-Up (*n* = 47)
VO_2peak_ (mL/kg/min)	15.3 ± 3.7	16.5 ± 4.2	14.8 ± 3.6	17.5 ± 4.7 **	14.2 ± 2.7	18.9 ± 4.7
Predicted VO_2peak_ (%)	56.4 ± 13.9	62.5 ± 14.4 *	54.0 ± 14.5	65.4 ± 16.0 **	50.5 ± 21.9	68.7 ± 19.1
Watt Max (W)	72.8 ± 25.1	80.6 ± 31.6	78.2 ± 24.9	86.8 ± 28.9	63.0 ± 7.0	102.8 ± 30.7
VE/VCO_2slope_	33.4 ± 6.2	32.2 ± 5.9	32.3 ± 5.6	31.3 ± 7.1	38.1 ± 3.8	28.9 ± 5.2 **
RER	1.14 ± 0.12	1.13 ± 0.09	1.13 ± 0.07	1.16 ± 0.11	1.07 ± 0.10	1.15 ± 0.10
HR Rest (bpm)	72.4 ± 10.8	68.7 ± 7.2	70.9 ± 10.9	68.3 ± 9.1	80.5 ± 27.6	69.4 ± 10.1
HR Peak (bpm)	106.6 ± 18.2	106.7 ± 19.9	106.3 ± 19.3	110.3 ± 15.9	126.0 ± 26.9	115.1 ± 22.1
HRR (bpm)	11.4 ± 9.5	14.1 ± 10.9	12.2 ± 11.2	15.0 ± 9.9	23.0 ± 2.8	18.7 ± 10.0
AT-VO_2_ (mL/kg/min)	11.4 ± 2.9	12.3 ± 2.9	11.4 ± 2.3	12.2 ± 3.7	10.9 ± 2.7	13.0 ± 2.9
AT-VO_2_ (%)	40.9 ± 11.5	45.7 ± 13.5	46.0 ± 13.4	45.8 ± 13.4	41.0 ± 11.3	50.2 ± 12.5 **
AT-Watt (W)	56.1 ± 24.8	57.2 ± 25.6	59.5 ± 19.7	55.1 ± 19.2	48.0 ± 21.3	67.3 ± 22.9 **
O_2_-Pulse (mL/beat)	11.4 ± 3.0	12.2 ± 3.5	12.6 ± 3.2	13.2 ± 3.8	10.2 ± 2.4	14.4 ± 3.8 *
ΔVO_2_/Δwork (mL/min/watt)	9.2 ± 1.6	9.6 ± 1.8	9.0 ± 1.7	10.1 ± 1.5 **	9.5 ± 0.6	10.1 ± 1.5
Peak Ventilation (L/min)	45.8 ± 10.6	55.0 ± 17.9 **	52.7 ± 14.7	56.4 ± 15.0	47.2 ± 9.5	63.6 ± 18.8 **
TIDAL volume (mL/kg)	1.5 ± 0.38	1.7 ± 0.56 *	1.7 ± 0.47	1.6 ± 0.44	1.6 ± 0.17	1.9 ± 0.5
Respiratory Rate (breaths/min)	30.8 ± 6.3	33.2 ± 6.9	31.7 ± 6.5	34.7 ± 6.0 *	30.0 ± 2.8	32.9 ± 7.0 **

Captions: * *p* < 0.05, ** *p* < 0.001 vs. baseline; ARNI_1_: angiotensin receptor neprilysin inhibitor (ARNI) dose of 24/26 mg bid; ARNI2: ARNI dose of 49/51 mg bid; ARNI_3_: ARNI dose of 97/103 mg bid; AT: anaerobic threshold; RER: respiratory exchange ratio; VE: ventilation; VE/VCO2slope: the slope of increase in ventilation over carbon dioxide output; VO2: oxygen consumption; HR: heart rate; HRR: heart rate recovery.
